# *N*-Step Pre-Training and Décalcomanie Data Augmentation for Micro-Expression Recognition

**DOI:** 10.3390/s22176671

**Published:** 2022-09-03

**Authors:** Chaehyeon Lee, Jiuk Hong, Heechul Jung

**Affiliations:** Department of Artificial Intelligence, Kyungpook National University, Daegu 41566, Korea

**Keywords:** deep learning, image processing, facial micro-expression, emotion recognition, convolutional neural network (CNN)

## Abstract

Facial expressions are divided into micro- and macro-expressions. Micro-expressions are low-intensity emotions presented for a short moment of about 0.25 s, whereas macro-expressions last up to 4 s. To derive micro-expressions, participants are asked to suppress their emotions as much as possible while watching emotion-inducing videos. However, it is a challenging process, and the number of samples collected tends to be less than those of macro-expressions. Because training models with insufficient data may lead to decreased performance, this study proposes two ways to solve the problem of insufficient data for micro-expression training. The first method involves *N*-step pre-training, which performs multiple transfer learning from action recognition datasets to those in the facial domain. Second, we propose Décalcomanie data augmentation, which is based on facial symmetry, to create a composite image by cutting and pasting both faces around their center lines. The results show that the proposed methods can successfully overcome the data shortage problem and achieve high performance.

## 1. Introduction

**Figure 1:**
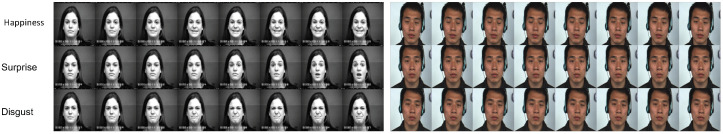
Examples of macro-expression (**left**) and micro-expression (**right**). Macro-expression samples have high intensity, while micro-expression samples show little change in facial expression.

Humans reveal personal feelings, intentions, and emotional conditions through their facial expressions. Generally, a person reveals emotions through explicit macro-expressions that last between 0.25 and 4 s. During these periods, the emotions expressed on the face and the actual feelings felt coincide. Conversely, when a person unconsciously reveals a hidden emotion in fractional time (e.g., 0.25 s), it is considered to be a micro-expression. These are likely to be missed or misinterpreted, even in laboratory settings. Figure 1 shows a comparison between the micro- and macro-expressions.

In 1966, Haggard et al. [1] first proposed the concept of micro-expressions. About three years later, Ekman et al. [2] witnessed this phenomenon while researching lie detection using interview videos of psychologists and patients. Robust micro-expression recognition systems are used in various fields, such as criminal recognition, lie detection, and psychological diagnosis. Owing to the broad applicability of micro-expression recognition, many studies have been conducted in recent years. These studies are primarily divided into hand-crafted feature-based methods [3,4,5,6,7,8] and deep learning-based methods [9,10,11,12,13,14]. The hand-crafted feature extraction was widely used at the beginning of the development of micro-expression detection technology because it extracts direct-designed features according to the given learning method.

On the other hand, the deep learning-based method automatically extracts features to solve specific tasks. Because such models are more convenient and achieve higher performance than hand-crafted feature-based methods, more studies using deep learning-based methods have recently emerged. Although micro-expression samples are limited and have a low intensity which causes difficulties, deep learning-based networks, such as convolutional neural networks (CNNs) and long short-term memory models, have achieved excellent progress.

Even though micro-expression datasets [4,15,16] consist of sequential frames, most studies thus far have used static images or optical flows [13,17,18,19], and few have used the frame sequence as input to the network [9,20]. Additionally, some studies have simultaneously used macro-expression data for learning with sequential inputs. However, to the best of our knowledge, no studies have transferred a model pre-trained on other more extensive datasets, such as action recognition datasets, to micro-expression datasets several times.

Therefore, in this paper, we propose a novel framework to overcome the insufficient number of clips in the micro-expression dataset. Our main contributions can be summarized as follows:

1. *N*-step pre-training is proposed which use extensive datasets in addition to facial datasets;
2. We propose a new augmentation method specialized for facial data, called “Décalcomanie”;
3. The combination of *N*-step pre-training and Décalcomanie augmentation outperforms state-of-the-art approaches in micro-expression recognition.

The remainder of this paper is organized as follows: Section 2 describes the related studies on micro-expression recognition. Section 3 describes the data preprocessing methods prior to training and the details of the proposed approach. We present the experimental settings and results in Section 4, and we conclude this paper in Section 5.

## 2. Related Work

In facial expression recognition, two methods are mainly used: hand-crafted feature-based methods and deep learning-based methods. In the hand-crafted feature extraction, LBP variants-based methods [3,4] and optical flow-based methods [13,19] are widely used. However, because micro-expression images have little movement and implied change, it is difficult for the hand-crafted feature-based method to achieve high performance.

Deep learning-based methods have performed better than hand-crafted feature-based methods in the field of computer vision in recent years. Therefore, studies using the deep learning-based method have been actively proposed even in micro-expression recognition. Wu et al. [9] used a three-stream combining 2D and 3D convolutional neural network to classify expressions and proposed two variants: intermediate fusion and late fusion. Xia et al. [10] improved the performance by adversarial learning using both the micro-expression dataset and the macro-expression dataset. Recently, Hung et al. [20] proposed multilevel transfer learning for a macro-expression dataset. They used related domain datasets for pre-training and performed experiments on the macro-expression dataset.

In this paper, we also propose a deep learning-based micro-expression recognition method. The methods mentioned earlier have not adequately utilized the pre-trained model and are dependent on additional information such as landmarks and optical flow. Therefore, we propose a novel method that uses a pre-trained model that learns large amounts of data across the *N*-step and a transformation method specialized in facial datasets called Decalcomanie. We emphasize that we utilize massive datasets with unrelated domains to facial in multistep pre-training, although Hung et al. [20] only utilized relevant domain datasets.

## 3. Proposed Method

**Figure 2:**
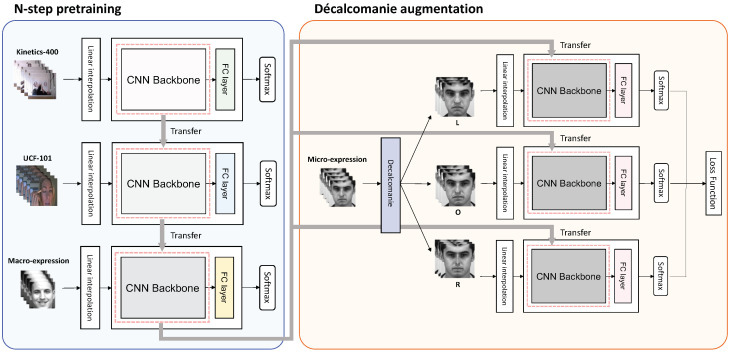
Framework of our proposed method (three-step pre-training with Décalcomanie augmentation). First, we train the model from scratch on Kinetics-400 and transfer it to UCF101. Next, we train the model on the macro-expression dataset. Finally, the model is fine-tuned on the micro-expression dataset by applying the Décalcomanie augmentation method. In each step, we replace the extant fully-connected (FC) layer of the model with a randomly initialized FC layer. *CNN,* convolutional neural network.

Insufficient data may lead to poor model performance. This section presents two methods to overcome the problem: *N*-step pre-training and Décalcomanie data augmentation. Before explaining the proposed methods in detail, we first show the preprocessing methods used to deal with the sequential data. The framework of the proposed method is shown in Figure 2.

### 3.1. Preprocessing

#### 3.1.1. Oversampling with Synthetic Samples

The micro-expression datasets have an imbalanced distribution. For example, in the Spontaneous Micro-expression (SMIC) dataset, the negative class has 70 samples, the positive class has 51, and the surprise class has 43. Training a model with such an imbalanced data may lead to overfitting. To handle this problem, Wu et al. [9] used an extension of the synthetic minority oversampling technique (SMOTE) [21] algorithm. They modified the SMOTE algorithm by using two samples to generate a new one using the following formula:

(1) $$x_{new,i}=\lambda \cdot x_{1,i}+\left(1-\lambda \right)\cdot x_{2,i},$$

where $x_{1,i}$ and $x_{2,i}$ are random samples belonging to the same category, and $x_{1,i}$ indicates the *i*-th frame of the sequence of sample $x_{1}$. λ is sampled from a uniform distribution (0, 1). They attempted to solve the class imbalance by creating a new sample belonging to the insufficient class using the algorithm. We also adopted this method to alleviate the imbalance problem.

#### 3.1.2. Frame Interpolation

The micro-expression dataset comprises sequence samples having different numbers of frames. Before training, all frames of the samples must be fixed at the same number to capture the appropriate temporal information. We adopted a linear interpolation method, provided by PyTorch, to fix the number of frames. The method samples down or up to the desired size through linear interpolation. We used this method because it is easy to implement, and there is no redundancy or loss of information.

### 3.2. N-Step Pre-Training

Transfer learning uses a pre-trained model to solve another task. Many studies have attempted to improve performance by transferring a pre-trained model that learned macro-expression data first to another micro-expression case. However, to the best of our knowledge, no studies have applied multistep transfer learning using massive sequential datasets that have even unrelated domains for micro-expression datasets (e.g., Kinetics-400 [22], UCF101 [23]), and a large-scale visual recognition dataset (e.g., ImageNet [24]). Although the micro-expression dataset has a different domain from the action recognition dataset, it is the same in that it contains temporal and spatial information. Therefore, we assumed that, if the model learns this helpful information first, there will be a benefit in the micro-expression data.

**Figure 3:**
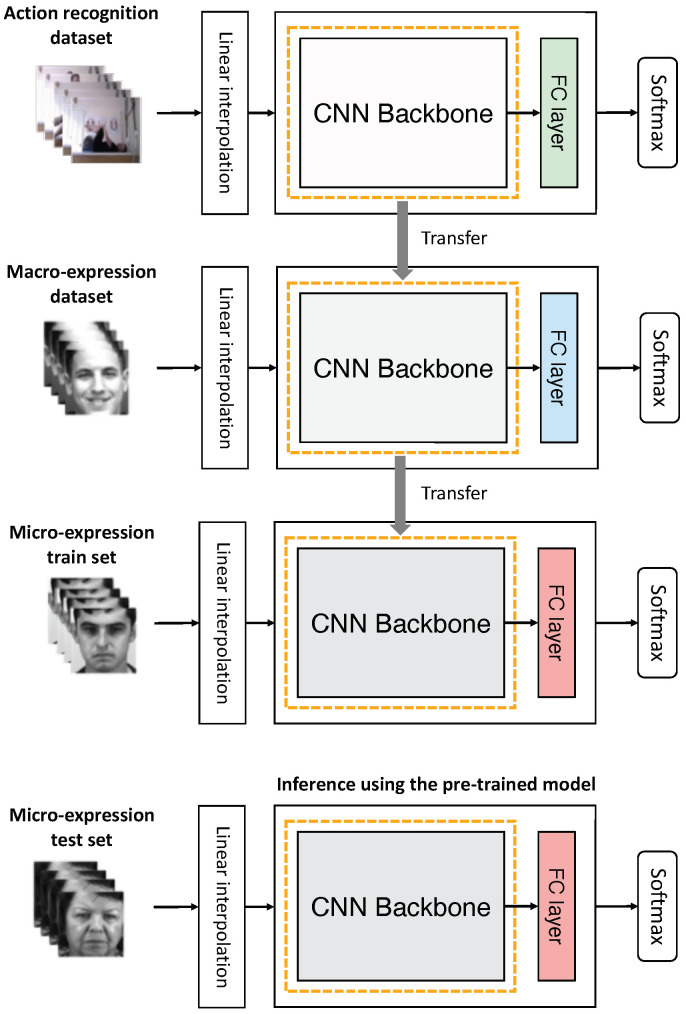
Procedure of two-step pre-training. We trained the model with the massive action recognition dataset, initialized the FC layer, and transferred the model to the macro-expression dataset. The pre-trained model was fine-tuned to the micro-expression training set again and then evaluated against the test set to obtain the final performance. *CNN*, convolutional neural network.

Multistep pre-training is composed of multiple-transfer learning. With one dataset, we can train the model from scratch and replace the fully-connected layer with a randomly initialized one. We can then fine-tune the model again on another dataset. A method of performing such transfer learning multiple times on different datasets is the multistep pre-training method that we propose. However, we emphasize that *N*-step pre-training does not simply attempt to implement multiple transfer learning; the sequence of datasets used in multiple steps is important. Figure 3 shows the procedure of two-step pre-training. Here, we train the model on the action recognition dataset first and then on the macro-expression dataset again. Lastly, we fine-tune the model on the micro-expression dataset.

### 3.3. Décalcomanie Augmentation

**Figure 4:**
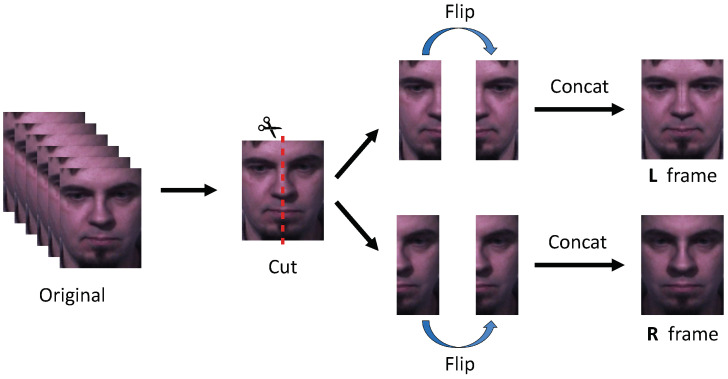
Procedure of Décalcomanie augmentation. First, we cut all the frames in half. We flip the cut left and right face frames, respectively, and concatenate the half-frame before flipping it to create new frames. We denote the new frame made of only the left faces and the new frame made of only the right faces as the L and R frames, respectively.

According to Mandal et al. [25], the left side of the human face is more expressive of middle-intensity happiness and minimum-intensity happiness and sadness. The right side is more expressive in the most intense expressions of happiness and sadness. Because both sides of the face reveal slightly different information about emotion, we devised Décalcomanie augmentation. Originally, Décalcomanie was a drawing technique that draws only half of a picture, folds it, and transcribes it to the other side, similar to the stamping principle. Décalcomanie augmentation also divides the face in half and transcribes back to the other side to create a new sample. Figure 4 illustrates the procedure of Décalcomanie augmentation.

As the video clips of micro-expression datasets used in this paper are not inverted, as when looking in a mirror, the left face and right face of the subject are originally right-side and left-side, respectively. However, to facilitate understanding and avoid confusion, we refer to the left and right faces as the left and right sides when looking at the video clip. If Décalcomanie is applied, the left- and right-side frames can be obtained alongside the original ones. Because three kinds of the frames can be obtained, a new training process was required to learn the additional samples. Therefore, we propose two versions of training process: (1) shared backbone and multiple losses, and (2) fusion with shared backbone.

#### 3.3.1. Shared Backbone and Multiple Losses

**Figure 5:**
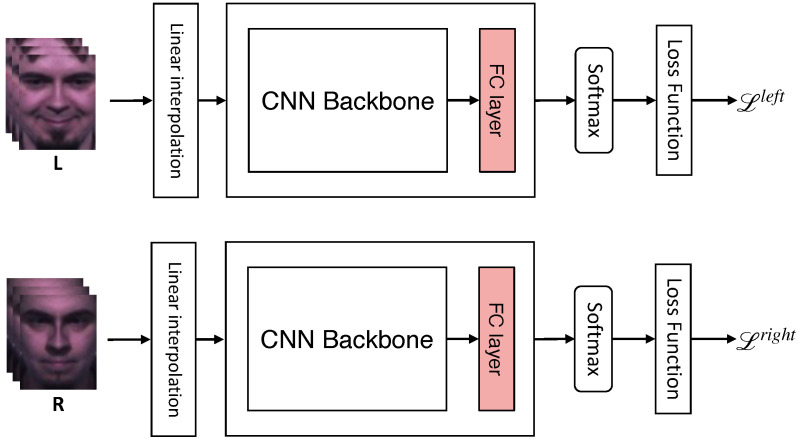
Procedure of “shared backbone and multiple losses” version when using LR frames. Each input frame shares the network and obtains each loss for each input frame. The training cost is calculated by combining each loss.

This version shares the backbone network and adds up the losses obtained by feed-forwarding each frame to the model. We used four subset cases consisting of **OLR**, **OL**, **OR**, and **LR**, where **O**, **L**, and **R** denote the original, left-side, and right-side frames, respectively. For each case, we calculated the training loss using Equation (2):

(2) $$\begin{array}{cc} L_{OLR} & =\lambda_{O}\cdot L^{original}+\lambda_{L}\cdot L^{left}+\lambda_{R}\cdot L^{right},\\ L_{OL} & =\lambda_{O}\cdot L^{original}+\lambda_{L}\cdot L^{left},\\ L_{OR} & =\lambda_{O}\cdot L^{original}+\lambda_{R}\cdot L^{right},\\ L_{LR} & =\lambda_{L}\cdot L^{left}+\lambda_{R}\cdot L^{right}, \end{array}$$

where L is cross-entropy loss. $\lambda_{O},\lambda_{L}$, and $\lambda_{R}$ are hyperparameters multiplied by each corresponding loss, and we set the sum of each λ in each equation to one. Figure 5 illustrates the procedure for using **LR** frames.

In this version, we tested the model’s performance with two cases: (1) using only the original frames and (2) using all frames as input. In the latter case, softmax(·) was applied after summing the output values obtained by feed-forwarding each input to the shared backbone.

#### 3.3.2. Fusion with Shared Backbone

**Figure 6:**
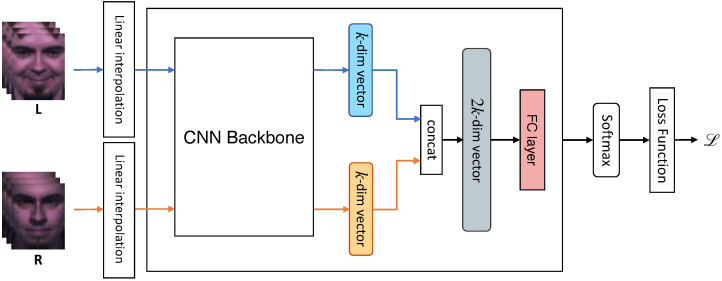
Procedure of “fusion with the shared backbone” version when using LR frames. Each input was forwarded to the shared backbone network, and we obtained a k-dimensional vector for each. After concatenating all vectors, we fed-forward the representation vector to calculate the training loss. *CNN,* convolutional neural network; *FC*, fully-connected.

Each feature extracted from the shared backbone was concatenated and forwarded to a single linear layer or multilayer perceptron (MLP). We set the hidden dimensions of the MLP to 256. Like the shared backbone and multiple losses version, we used **OLR**, **OL**, **OR**, and **LR**, which are possible input cases, and the combination of the frames used during training was the same for testing. Figure 6 shows the procedure of fusion with the shared backbone using **LR** frames.

## 4. Experiments

First, we summarize the experimental setup and then verify the effectiveness of each proposed method. Lastly, we show the performance of the model when the two proposed methods are combined.

### 4.1. Setup

Here, we summarize the dataset details used in the experiment, the metrics used to evaluate the model performance, and the implementation details in order.

#### 4.1.1. Datasets

##### Facial Dataset

**Figure 7:**
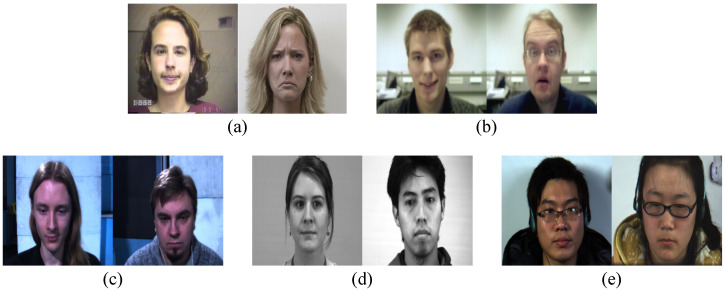
Samples of macro- and micro-expression datasets. CK+ and Oulu-CASIA are macro-expression datasets, and SMIC, CASME2, and SAMM are micro-expression datasets. (**a**) CK+; (**b**) Oulu-CASIA; (**c**) SMIC; (**d**) CASME2; (**e**) SAMM.

Facial expressions can be divided into two main categories: macro-expression and micro-expression. Macro-expressions appear on our faces without any oppression when emotions are usually expressed. Macro-expressions are easy to recognize because of their long duration (0.25–4 s), and the emotion to be revealed is clearly expressed. Examples of each dataset we used are shown in Figure 7. The details of the macro-expression datasets are as follows:

- **Extended Cohn–Kanade (CK+)** [26] initially contained 593 video sequences of 123 subjects aged 18 to 50 with diverse genders and heritage. The samples were collected at 30 fps with a resolution of 640 × 490 or 640 × 480 pixels. These samples were divided into seven categories: anger, disgust, fear, sadness, contempt, happiness, and surprise.
- **Oulu-CASIA** [27] consists of six emotion classes (i.e., anger, disgust, fear, sadness, happiness, and surprise) from 80 people between 23 and 58 years old. The camera recorded the expressions at 25 fps with a resolution of 320 × 240 pixels. This dataset was collected under two conditions: near-infrared (NIR) and visible (VIS) light systems. VIS images consist of three different versions: dark, strong, and weak. The dark versions are created when samples are taken in a dark environment. The strong version is the case where the emotion of the subject’s expression stands out, and the weak version is the opposite.

Micro-expressions, alternatively, are unconsciously short facial expressions typically made under stress. They appear for only 0.5 s and are very subtle, making it difficult to judge emotions, even when intentionally observed. There are many micro-expression datasets, but high quality ones include SMIC [4], CASME2 [15], and SAMM [16]. These datasets were recorded in a laboratory environment, and subjects were asked to maintain a “poker face” without showing emotions as much as possible under different stimuli. The details of each dataset are as follows:

- **SMIC** [4] consists of samples recorded by 100 fps high-speed cameras and samples recorded at standard speed with 25 fps of both VIS and NIR light ranges. Each subset is referred to as SMIC-HS, SMIC-VIS, and SMIC-NIR. The SMIC-HS consists of 164 samples taken from 16 subjects, each assigned a label of “negative”, “positive”, or “surprise”.
- **CASME2** [15] is an improved version of the existing micro-expression dataset, CASME [28]. CASME2 was filmed using a high-speed 250 fps camera and crops only the face part at a size of 280 × 340 pixels. The dataset has 247 samples generated from 26 subjects, but some expressions are unevenly distributed because they were difficult to derive under laboratory conditions. It provides five classes of micro-expressions.
- **SAMM** [16] comprises 159 samples collected from 32 participants. Although other datasets lack ethnic diversity, the SAMM dataset consists of 13 different ethnicities; the average age was 33.24 years, and the gender distribution was almost identical. Each sample was collected at a high speed of 200 fps with a high resolution of 2040 × 1088. The labels for each sample were emotion labels designated by participants through surveys.

##### Action Recognition Dataset

Because the facial dataset is sequential, the model learns spatial and temporal information during training. The final model performance can be higher if a large-scale action recognition dataset is used to learn spatiotemporal information in advance before learning the facial dataset.

- **Kinetics-400** [22] consists of 400 human action classes and has at least 400 video clips per class. These videos were taken from YouTube and focus on human actions. The dataset covers a broad range of classes that are largely divided into person (e.g., drawing, laughing, and fist-pumping), person-person (e.g., hugging and shaking hands), and person–object actions (e.g., mowing the lawn and washing dishes). Unlike facial datasets, the entire human body is often displayed on a video.
- **UCF-101** [23] consists of 101 action categories and videos collected from YouTube. It contains 13,320 videos and is one of the most challenging datasets because the videos were filmed under significant variations of camera motion. The categories are largely classified into five types: human–object interaction, body motion only, human–human interaction, playing music instruments, and sports. Similar to Kinetics, UCF-101 is a human action recognition dataset, but the proportion of videos capturing face or upper body is slightly higher.

#### 4.1.2. Evaluation Metrics

In this paper, we use the leave-one-subject-out (LOSO) cross-validation protocol to evaluate the model. We adopted this protocol to make up for the micro-expression dataset having a biased number of samples per subject. If *K* subjects are in the dataset, the LOSO protocol divides the experiment into *K* folds. It uses one subject as the test set and the remaining K−1 subjects as the training set, which leads to *K* experiments. Meanwhile, the micro-expression datasets also have a biased distribution of samples for emotion classes, providing an imbalanced distribution. To reduce the potential bias, we used weighted average recall (WAR), unweighted average recall (UAR), and unweighted F1 score (UF1) as evaluation standards.

(3) $$\mathrm{UF}1=\frac{1}{C}\sum_{c=1}^{C}{F1}_{c},\;\mathrm{UAR}=\frac{1}{C}\sum_{c=1}^{C}\frac{TP_{c}}{N_{c}},\;\mathrm{WAR}=\frac{\sum^{C}TP_{c}}{\sum_{c=1}^{C}N_{c}}$$

where

(4) $$F1=\frac{2TP_{c}}{2TP_{c}+FP_{c}+FN_{c}}$$

In Equation (3), *C* is the number of classes, and TP, FN, and FP are the true positive, false negative, and false positive, respectively. $N_{c}$ is the total number of samples in category *C*.

#### 4.1.3. Implementation Details

We used one NVIDIA RTX A6000 48 GB GPU per experiment. For *N*-step pre-training experiments, we trained 3D-ResNet-50 [29] for 30 epochs using Adam [30] with $\beta_{1}=0.9$, $\beta_{2}=0.999$, a batch size of 30, and a learning rate of 0.0001, which decayed by 10 at the 13th, 18th, and 22nd epoch. We resized all frames to 112. In the case of Décalcomanie experiments, we trained 3D-ResNeXt-101 [29] for 100 epochs. This is different in that the learning rate is decayed by 10 at the 30th, 60th, and 80th epochs; however, all other hyperparameters are the same as in the pre-training experiment. In the case of **OLR** frames, we set $\lambda_{O}$, $\lambda_{L}$, and $\lambda_{R}$ to 0.4, 0.4, and 0.2, respectively. For **OL**, **OR**, and **LR**, we set λ to 0.5. We used synthetic samples generated using extended SMOTE with the *N*-step pre-training experiments, but not in the Décalcomanie experiments. Because synthetic samples have mixed faces, cutting them in half and combining them can cause noisy representations. Furthermore, because the number of frames in each video sample of the micro-expression datasets is different, it was necessary to fix the datasets’ frames to capture the temporal information. We used the linear interpolation to set the number of frames to their average number. As a result, we set the video lengths of SMIC, SAMM, and CASME2 to 34, 74, and 66, respectively.

### 4.2. Effect of N-Step Pre-Training

**Figure 8:**
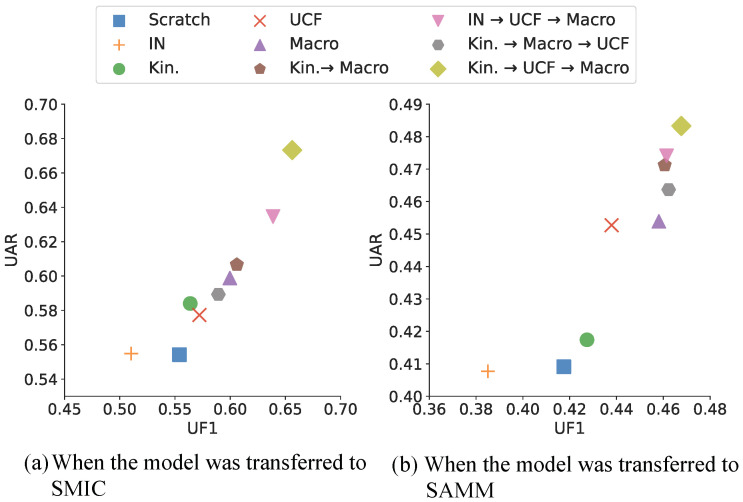
Visualization of *N*-step pre-training performance (UF1 and UAR). In both cases, the performance of Kinetics → UCF → Macro achieves the best performance (Kin.: Kinetics-400).

We conducted an *N*-step pre-training experiment for a micro-expression dataset using 3D-ResNet-50. The datasets used as source datasets were Kinetics-400, UCF101, ImageNet, and macro-expression datasets CK+ and Oulu-CASIA. As Kinetics-400 and ImageNet are massive, we use public pre-trained models on those without direct learning to reduce learning costs. We used SMIC and SAMM as target datasets in this experiment. We showed a visual comparison of the UF1 and UAR performance of each *N*-step pre-trained model in Figure 8. We also presented the numerical results in Table 1. For convenience, We refer to ImageNet, Kinetics-400, UCF101, and a combination of CK+ and Oulu-CASIA as IN, Kinetics, UCF, and Macro, respectively. We analyzed the result with a particular focus on UF1 and UAR. Through an experimental investigation, we found three interesting results.

First, in one-step pre-training, we found that the model pre-trained on IN performed worse than the scratch model, and in the case of the rest of the datasets, they performed better, and their performance was higher in the order of kinetics, UCF, and Macro. Although IN is large-scale, it hinders model performance because it is composed of still images without temporal information. In addition, Kinetic has a higher proportion of frames focusing on the whole body, and UCF has a higher proportion of frames with the upper body and face than Kinetics. Hence, UCF is slightly closer than Kinetics, although its domain is clearly different from the facial dataset. Therefore, we assumed for that reason that the performance of the model pre-trained on UCF was higher than that of the model pre-trained on Kinetics. For macro-expression datasets with a similar domain to micro-expression datasets, the performance was the highest among one-step pre-training methods.

Second, we analyzed whether the order of datasets used for pre-training affects performance in *N*-step pre-training. In Table 1, the last second lines show the experimental results obtained by switching UCF and Macro with the dataset learned in the last step. Training the model on Macro in the last step was better than UCF, suggesting that the dataset order of the pre-training process could achieve higher performance as it proceeded in the order of datasets with domains like the micro-expression domain.

Finally, when the macro-expression dataset was used for pre-training in the last step, we compared the Scratch, Macro, Kinetics → Macro, IN → UCF → Macro, and Kinetics → UCF → Macro to evaluate whether performance improves as the number of steps increases. As a result, we confirmed that, with more steps, performance improved. Furthermore, when comparing IN → UCF → Macro and Kinetics → UCF → Macro, which used the same three-step pre-training, the performance of Kinetics → UCF → Macro was better than that of IN → UCF → Macro, which is presumed to have been caused by the presence or absence of temporal information, as mentioned.

### 4.3. Effect of Décalcomanie Augmentation

We trained 3D-ResNeXt-101 from scratch without using the pre-trained model solely to check the performance of Décalcomanie data augmentation. We did not use the aforementioned oversampling method. The datasets used in experiments are SMIC, CASME II, and SAMM. We resized each frame to 112 and applied scale, rotation, and horizontal flip augmentation in addition to Décalcomanie.

The results of the shared backbone and multiple loss on SMIC, SAMM, and CASME II using 3D-ResNeXt-101 from scratch are shown in Table 2. The first line of the table means the result when we did not apply Décalcomanie augmentation. First, we tested if Décalcomanie augmentation can be used as test time augmentation. During training time, we only used the original frame as input and used various frames created via Décalcomanie such as **OLR**, **OL**, and **OR** during test time. The top four lines of Table 2 prove that Décalcomanie can be utilized as test time augmentation. The performances got higher for every dataset when our proposed augmentation method was used as test time augmentation. For example, when test time augmentation was not used for the SMIC dataset, UF1, UAR, and WAR were 0.5833, 0.5680, and 0.5671, respectively. However, UF1, UAR, and WAR rose to 0.5985, 0.5929, and 0.6098 when OLR frames created using the Décalcomanie augmentation were input during the test, meaning that the performance was increased by 0.0152, 0.0246, and 0.0427, respectively.

Then, we also conducted experiments to verify the effect of Décalcomanie as data augmentation during training. In most of the experiments, models with Décalcomanie achieved higher performance than those without it. In addition, overall, the model with a single linear layer obtained higher performance than the model with a multilayer perceptron. In the experiments using SMIC and CASME II, the performance improvement was the highest in the sub-experiment using **OLR** frames as input. We hypothesize that these results occurred because emotional expressions were well represented on both sides of the face. Unlike the results, in the case of SAMM, the performance improvement was the greatest when using the **OR** frames. When we compared SAMM to other datasets, the subjects’ expressions were significantly more emotionally inhibited. Although it is difficult to detect changes due to minimal facial changes, we assume that the reason for the highest performance when inserting the **OR** frames is that there was a little more minimum intensity on the right side of the subject’s face.

The results of the fusion with the shared backbone are shown in Table 3. Overall, the fusion with shared backbone version does not have a higher overall performance than the shared backbone and multiple losses version. However, there was a performance improvement compared to when Décalcomanie was not applied. Using a single linear as a classifier performed better than using multilayer perceptron in most cases. Due to the small number of data, the performance of the deeper model is not higher. In this experiment, UF1, UAR, and WAR were highest in SMIC and CASME II when **OL** or **OLR** frames were used, and in the case of SAMM, when **OR** frames were used achieved the highest performance. As can be seen from the results, it can be assumed that SMIC and CASME II contained facial expression information evenly on both faces. SAMM also showed more minor performance improvements than did the other two datasets because emotional expression was further suppressed.

To further research the effectiveness of the Décalcomanie method, we compared it with other augmentation methods. The results are shown in Table 4. First, we compared it with basic augmentation methods usually used. We rotated images with 30 degrees, resized images with scale (1.1,1.1), or flipped images horizontally. The results of applying primary augmentations show low performances, which means that the simple transformation of the frames does not have much effect. When we applied the SMOTE algorithm that produces the synthetic samples used by Wu et al. [9] with simple augmentations, the performance of all the metrics was over 0.6. However, when we applied our proposed augmentation method, Decalcomanie, the models’ performance was much higher than when using SMOTE. This result demonstrates that Décalcomanie has a remarkable effect on facial data.

### 4.4. Overall Results

Here, we combined the *N*-step pre-training and Décalcomanie data augmentation to evaluate the overall results. We used the best combination in the *N*-step pre-training experiment (Kinetics → UCF → Macro). 3D-ResNeXt-101 was used as the backbone network. After *N*-step pre-training, we fine-tuned the pre-trained model on the SMIC dataset and evaluated its performance.

Table 5 shows the results. As stated, shared backbone and multiple losses experiments had better performance than did fusion with shared backbone experiments. Earlier, the performance was highest on the SMIC dataset when experimenting with **OLR** or **OL** frames, showing the same results in this experiment. In the case of shared backbone and multiple losses, when both N-step pre-training and the Décalcomanie method were applied compared to Scratch, UF1, UAR, and WAR increased by +0.2104, +0.2267, and +0.2256, respectively. For fusion with shared backbone experiments, the performance was lower than shared backbone and multiple losses; however, the performance was still significantly better than scratch.

We compared our proposed method with other state-of-the-art approaches which used the LOSO protocol and the same number of classes for a fair comparison. Since the results of the shared backbone and multiple losses were better than the fusion with shared backbone in the previous experiments, we used the figures of the shared backbone and multiple losses version for comparison. Refs. [3,4,13,19] are hand-crafted feature-based methods and [10,31,32,33] are deep learning-based methods which used additional datasets in addition to the micro-expression dataset. The comparison with these methods is shown in Table 6. We achieve the best performance when our proposed methods, such as *N*-step pre-training and Décalcomanie data augmentation, are combined.

## 5. Conclusions

In this paper, we proposed *N*-step pre-training and Décalcomanie augmentation to avoid the data shortage problem in micro-expression recognition. In *N*-step pre-training, we transferred the model multiple times on various datasets in a specific order. Furthermore, we devised a data augmentation method specialized for the face called Décalcomanie. We evaluate the effectiveness of each method on micro-expression datasets. When combining *N*-step pre-training and Décalcomanie augmentation, the experimental results show that our proposed methods outperform the state-of-the-art methods on the SMIC dataset.

## Figures and Tables

**Table 1:** Experimental results of *N*-step pre-training (IN: ImageNet, Kinetics: Kinetics-400, UCF: UCF101, Macro: a combination of CK+ and Oulu-CASIA). (**a**) When training the 3D-ResNet-50 on the SMIC. (**b**) When training the 3D-ResNet-50 on the SAMM.

(a)					(b)				
Pre-Trained	Step	UF1	UAR	WAR	Pre-Trained	Step	UF1	UAR	WAR
Scratch	0	0.5540	0.5542	0.5549	scratch	0	0.4175	0.4091	0.5515
IN	1	0.5104	0.5549	0.5122	IN	1	0.3850	0.4077	0.4412
Kinetics	1	0.5638	0.5840	0.5610	Kinetics	1	0.4274	0.4174	**0.5588**
UCF	1	0.5721	0.5773	0.5793	UCF	1	0.4379	0.4527	0.5441
Macro	1	0.5997	0.5988	0.6098	Macro	1	0.4581	0.4539	0.5294
Kinetics → Macro	2	0.6060	0.6066	0.5976	Kinetics → Macro	2	0.4606	0.4712	0.5441
IN → UCF → Macro	3	0.6388	0.6346	**0.6585**	IN → UCF → Macro	3	0.4614	0.4741	**0.5588**
Kinetics → Macro → UCF	3	0.5893	0.5893	0.5915	Kinetics → Macro → UCF	3	0.4623	0.4637	0.5515
Kinetics → UCF → Macro	3	**0.6561**	**0.6733**	0.6463	Kinetics → UCF → Macro	3	**0.4677**	**0.4833**	0.5515

**Table 2:** Experimental results of Décalcomanie (Shared Backbone and Multiple Losses). (**a**) When training the 3D-ResNeXt-101 on the SMIC. (**b**) When training the 3D-ResNeXt-101 on the CASME II. (**c**) When training the 3D-ResNeXt-101 on the SAMM.

(a)					(b)					(c)				
Train	Test	UF1	UAR	WAR	Train	Test	UF1	UAR	WAR	Train	Test	UF1	UAR	WAR
O	O	0.5833	0.5680	0.5671	O	O	0.4586	0.4535	0.5181	O	O	0.4967	0.4994	0.6066
	OLR	0.5985	0.5929	0.6098		OLR	0.5144	0.5144	0.5382		OLR	0.5333	0.5518	0.6177
	OL	0.5856	0.5798	0.6159		OL	0.4973	0.4987	0.5261		OL	0.5160	0.5393	0.5882
	OR	0.5884	0.5918	0.5915		OR	0.4809	0.4875	0.5141		OR	0.5415	0.5609	0.6103
OLR	O	0.6242	**0.6454**	0.6220	OLR	O	**0.5581**	**0.5527**	**0.6104**	OLR	O	0.5413	0.5419	0.6103
	OLR	**0.6441**	0.6369	**0.6524**		OLR	0.4607	0.4672	0.5783		OLR	0.4729	0.4653	0.5809
OL	O	0.6114	0.6352	0.6098	OL	O	0.5165	0.5229	0.5944	OL	O	0.5647	0.5580	0.6471
	OL	0.6225	0.6326	0.6220		OL	0.5432	0.5422	**0.6104**		OL	0.5699	0.5668	0.6471
OR	O	0.6283	0.6303	0.6280	OR	O	0.4809	0.4882	0.5622	OR	O	0.5276	0.5265	0.6250
	OR	0.6293	0.6125	0.6280		OR	0.5018	0.4987	0.5663		OR	**0.6011**	**0.6157**	**0.6691**
LR	O	0.6150	0.6125	0.6159	LR	O	0.5419	0.5399	0.6064	LR	O	0.5169	0.5173	0.5956
	LR	0.6227	0.6364	0.6220		LR	0.4933	0.4920	0.5783		LR	0.5087	0.4989	0.6029

**Table 3:** Experimental For the MLP column, × denotes that a single fully-connected layer is used for the classfier. results of Décalcomanie (Fusion with Shared Backbone). (**a**) When training the 3D-ResNeXt-101 on the SMIC. (**b**) When training the 3D-ResNeXt-101 on the CASME II. (**c**) When training the 3D-ResNeXt-101 on the SAMM. “×” in the MLP column indicates that a single fully-connected layer is used for the classifier.

(a)					(b)					(c)				
Train /Test	MLP	UF1	UAR	WAR	Train /Test	MLP	UF1	UAR	WAR	Train /Test	MLP	UF1	UAR	WAR
O	×	0.5833	0.5680	0.5671	O	×	0.4586	0.4535	0.5181	O	×	0.4967	0.4994	0.6066
	◯	0.5509	0.5507	0.5549		◯	0.4235	0.4385	0.5181		◯	0.4712	0.4753	0.5662
OLR	×	**0.6346**	**0.6438**	**0.6463**	OLR	×	**0.4834**	**0.4974**	**0.5588**	OLR	×	0.5061	0.5159	0.6176
	◯	0.6068	0.6024	0.6220		◯	0.4800	0.4703	0.5582		◯	0.5093	0.5208	0.6029
OL	×	0.6060	0.6177	0.6098	OL	×	0.4697	0.4549	0.5261	OL	×	0.4943	0.5018	0.5368
	◯	0.6172	0.6197	0.6159		◯	0.4411	0.4549	0.5261		◯	0.4626	0.5152	0.5055
OR	×	0.6228	0.6261	0.6280	OR	×	0.4656	0.4720	0.5141	OR	×	**0.5938**	**0.6126**	**0.6691**
	◯	0.5866	0.5935	0.5915		◯	0.4559	0.4724	0.4940		◯	0.4895	0.4912	0.5809
LR	×	0.5917	0.5992	0.6037	LR	×	0.4536	0.4385	0.5422	LR	×	0.5005	0.5056	0.5735
	◯	0.5919	0.5856	0.6098		◯	0.4695	0.4675	0.5181		◯	0.5048	0.5450	0.5441

**Table 4:** Comparison between various data augmentation techniques on the SMIC dataset. *SBML,* Shared backbone and multiple losses; *FSB*, Fusion with a shared backbone.

Method	UF1	UAR	WAR
RandomRotation	0.5768	0.5781	0.5976
RandomResize	0.5734	0.5773	0.5731
RandomHorizontalFlip	0.5719	0.5766	0.5792
SMOTE [9] (+Rotation, Resize, Flip)	0.6063	0.6006	0.6220
**Décal-SBML (+Rotation, Resize, Flip)**	**0.6441**	**0.6369**	**0.6524**
**Décal-FSB (+Rotation, Resize, Flip)**	**0.6346**	**0.6438**	**0.6463**

**Table 5:** Experimental results when training 3D-ResNeXt-101 using both N-step pre-training and Décalcomanie on the SMIC dataset. (**a**) Shared Backbone and Multiple Losses. (**b**) Fusion with a shared backbone. “×” in the MLP column denotes that a single fully-connected layer is used for the classifier.

(a)					(b)				
Train	Test	UF1	UAR	WAR	Train/Test	MLP	UF1	UAR	WAR
Scratch		0.5833	0.5680	0.5671	O	×	0.5833	0.5680	0.5671
OLR	O	**0.7937**	**0.7947**	**0.7927**	OLR	×	**0.7566**	0.7554	**0.7561**
	OLR	0.7382	0.7411	0.7378		◯	0.7228	0.7209	0.7195
OL	O	0.7350	0.7542	0.7317	OL	×	0.7551	**0.7673**	0.7500
	OL	0.7156	0.7226	0.7134		◯	0.7043	0.7262	0.7012
OR	O	0.7858	0.7893	0.7805	OR	×	0.7262	0.7227	0.7317
	OR	0.7719	0.7828	0.7683		◯	0.7507	0.7506	**0.7561**
LR	O	0.7460	0.7548	0.7439	LR	×	0.7454	0.7470	0.7317
	LR	0.7205	0.7257	0.7195		◯	0.7341	0.7411	0.7317

**Table 6:** Comparison to other methods on the SMIC dataset. The performance of the proposed method achieved the highest value when both *N*-step pre-training and Décalcomanie were used (font in bold).

Method	UF1	UAR	WAR
LBP-TOP [4]	-	-	0.4878
STLBP-IP [3]	-	-	0.5951
Bi-WOOF [19]	0.6200	-	0.6220
3D flow-based CNN [13]	-	-	0.5549
TSCNN [31]	0.7236	-	0.7274
MTMNet [10]	0.7680	0.7440	-
SMA-STN [32]	0.7683	-	0.7744
MiNet [33]	0.7780	-	0.7860
**Proposed**	**0.7937**	**0.7947**	**0.7927**

## Data Availability

Not applicable.
